# Supplementary material for: A Low-Cost, Integrated Immunization, Health, and Nutrition Intervention in Conflict Settings in Pakistan—The Impact on Zero-Dose Children and Polio Coverage
Source: Pathogens. 2024 Feb 20;13(3):185. doi: 10.3390/pathogens13030185 (PMC10975858; doi:10.3390/pathogens13030185)
Supplement: Supplementary file 1 [file pathogens-13-00185-s001.zip › pathogens-2850642-supplementary.pdf]

Supplementary Material

Table S1: Peshawar - UC-Level Immunization Coverage at Baseline and Endline for Children Under-Three in Intervention and Control Areas

	**Baseline**		**Endline**		**Control-% diff (95% CI)**	**Intervention-% diff (95% CI)**	**DID estimate-% diff (95% CI)**	**DID estimate-P value**
	**Control**	**Intervention**	**Control**	**Intervention**				
**Immunization Status**								
	**N=333**	**N=350**	**N=364**	**N=300**				
Fully Immunized	136 (39.8)	161 (42.6)	222 (61.3)	208 (69.1)	21.5 (11.3, 31.7)	26.5 (15.4, 37.6)	5.0 (-9.7, 19.8)	0.4998
Partially Immunized	188 (57.2)	180 (54.9)	136 (36.9)	89 (29.3)	-20.3 (-30.1, -10.4)	-25.7 (-35.9, -15.4)	-5.4 (-19.3, 8.6)	0.4451
Non-recipients of Routine Immunization (NR-RI)	9 (3.0)	9 (2.5)	6 (1.7)	3 (1.6)	-1.2 (-4.3, 1.9)	-0.9 (-3.9, 2.2)	0.4 (-3.9, 4.6)	0.8701
Zero Dose	9 (3.0)	6 (1.5)	5 (1.5)	1 (0.5)	-1.5 (-4.4, 1.4)	-1.0 (-2.9, 0.8)	0.5 (-2.9, 3.8)	0.7865
Ever had Vaccination Card	279 (78.0)	305 (88.8)	351 (96.0)	280 (93.1)	18.0 (4.6, 31.4)	4.3 (-2.5, 11.1)	-13.7 (-28.4, 1.0)	0.0668
**At Birth**								
	**N=333**	**N=350**	**N=364**	**N=300**				
BCG	323 (96.4)	332 (94.6)	355 (97.4)	297 (98.4)	1.1 (-3.4, 5.5)	3.8 (-2.0, 9.7)	2.8 (-4.4, 9.9)	0.4466
OPV0	319 (95.7)	339 (97.0)	345 (94.9)	283 (93.4)	-0.7 (-5.3, 3.8)	-3.5 (-8.3, 1.2)	-2.8 (-9.2, 3.6)	0.3878
**At 6 Weeks**								
	**N=327**	**N=348**	**N=353**	**N=297**				
OPV1	183 (57.8)	203 (56.1)	318 (89.9)	271 (89.9)	32.1 (18.2, 46.0)	33.7 (20.0, 47.4)	1.6 (-17.5, 20.7)	0.8679
Penta 1	301 (91.3)	269 (75.6)	319 (90.1)	270 (89.4)	-1.3 (-7.3, 4.8)	13.8 (1.4, 26.3)	15.1 (1.6, 28.7)	0.0292
PCV 1	296 (90.4)	264 (73.8)	320 (90.4)	267 (88.7)	0.0 (-6.0, 6.1)	14.9 (1.7, 28.1)	14.9 (0.7, 29.1)	0.0404
Rota Virus 1	303 (91.6)	289 (81.8)	315 (89.1)	262 (87.3)	-2.5 (-8.7, 3.7)	5.5 (-5.0, 15.9)	7.9 (-3.9, 19.8)	0.1864
**At 10 Weeks**								
	**N=312**	**N=343**	**N=337**	**N=293**				
OPV 2	159 (51.6)	185 (51.9)	272 (81.0)	249 (85.2)	29.4 (14.7, 44.1)	33.4 (20.6, 46.1)	4.0 (-15.1, 23.0)	0.6796
Penta 2	166 (54.5)	197 (55.0)	281 (83.0)	254 (86.5)	28.5 (15.6, 41.4)	31.5 (17.4, 45.6)	3.0 (-15.8, 21.7)	0.7526
PCV 2	166 (54.5)	196 (54.7)	280 (82.9)	250 (85.2)	28.3 (15.5, 41.1)	30.5 (16.1, 44.9)	2.2 (-16.7, 21.0)	0.8201
Rota Virus 2	163 (53.4)	187 (52.2)	274 (80.7)	251 (85.6)	27.3 (14.1, 40.5)	33.4 (20.3, 46.6)	6.1 (-12.1, 24.3)	0.5050
**At 14 Weeks**								
	**N=301**	**N=337**	**N=323**	**N=284**				
OPV 3	145 (49.0)	169 (47.8)	235 (73.0)	235 (83.4)	24.1 (10.5, 37.7)	35.6 (24.8, 46.5)	11.6 (-5.5, 28.6)	0.1801
Penta 3	126 (41.5)	164 (45.9)	244 (75.7)	232 (81.2)	34.2 (23.8, 44.7)	35.3 (23.3, 47.3)	1.1 (-14.5, 16.7)	0.8891
PCV 3	126 (41.5)	164 (45.9)	233 (72.5)	231 (80.8)	31.0 (20.2, 41.8)	34.9 (23.1, 46.6)	3.9 (-11.8, 19.5)	0.6243
IPV 1	265 (88.9)	237 (67.7)	240 (74.3)	214 (74.8)	-14.6 (-22.0, -7.2)	7.0 (-7.4, 21.5)	21.7 (5.8, 37.5)	0.0081
**At 9 Months**								
	**N=248**	**N=256**	**N=259**	**N=233**				
Measles 1	199 (79.1)	191 (71.0)	205 (79.1)	183 (78.4)	0.0 (-7.7, 7.6)	7.4 (-5.5, 20.2)	7.4 (-7.2, 22.1)	0.3150
**At 15 months**								
	**N=202**	**N=204**	**N=195**	**N=177**				
Measles 2	113 (51.1)	125 (55.5)	139 (71.2)	123 (69.8)	20.1 (9.0, 31.2)	14.2 (-0.7, 29.2)	-5.9 (-24.1, 12.4)	0.5231

Table S2: Lakki Marwat - UC-Level Immunization Coverage at Baseline and Endline for Children Under-Three in Intervention and Control Areas

	Baseline		Endline		Control-% diff (95% CI)	Intervention-% diff (95% CI)	DID estimate-% diff (95% CI)	DID estimate-P value
	Control	Intervention	Control	Intervention				
**Immunization Status**								
	**N=321**	**N=345**	**N=293**	**N=363**				
Fully Immunized	22 (6.4)	25 (5.9)	51 (18.4)	110 (31.0)	11.9 (5.5, 18.3)	25.0 (16.7, 33.4)	13.1 (2.9, 23.3)	0.0127
Partially Immunized	73 (21.9)	67 (19.7)	130 (43.1)	208 (56.5)	21.3 (12.3, 30.2)	36.9 (27.6, 46.2)	15.6 (3.0, 28.2)	0.0157
Non-recipients of Routine Immunization (NR-RI)	226 (71.7)	253 (74.4)	112 (38.5)	45 (12.5)	-33.2 (-43.2, -23.2)	-61.9 (-70.3, -53.6)	-28.7 (-41.4, -16.1)	<0.0001
Zero Dose	226 (71.7)	251 (73.9)	27 (10.8)	11 (2.8)	-60.9 (-68.3, -53.5)	-71.1 (-79.8, -62.4)	-10.2 (-21.3, 0.9)	0.0714
Ever had Vaccination Card	67 (19.3)	63 (16.7)	180 (61.6)	301 (82.5)	42.2 (32.8, 51.7)	65.7 (57.7, 73.8)	23.5 (11.5, 35.5)	0.0002
**At Birth**								
	**N=321**	**N=345**	**N=293**	**N=363**				
BCG	90 (26.9)	90 (25.1)	176 (59.9)	313 (86.2)	33.0 (23.0, 43.1)	61.1 (52.5, 69.7)	28.1 (15.2, 40.9)	<0.0001
OPV0	90 (26.9)	84 (22.7)	161 (54.9)	288 (79.6)	28.0 (19.6, 36.5)	56.9 (47.0, 66.8)	28.9 (16.2, 41.5)	<0.0001
**At 6 Weeks**								
	**N=309**	**N=327**	**N=285**	**N=353**				
OPV1	72 (22.4)	60 (15.8)	160 (55.9)	279 (79.2)	33.5 (24.0, 43.0)	63.4 (54.4, 72.5)	29.9 (17.2, 42.7)	<0.0001
Penta 1	87 (26.9)	74 (20.5)	176 (61.4)	306 (86.6)	34.5 (24.6, 44.4)	66.1 (57.0, 75.2)	31.6 (18.5, 44.7)	<0.0001
PCV 1	82 (25.2)	73 (20.0)	176 (61.4)	305 (86.4)	36.3 (26.3, 46.2)	66.4 (57.3, 75.4)	30.1 (17.0, 43.2)	<0.0001
Rota Virus 1	75 (22.8)	72 (19.9)	176 (61.4)	306 (86.6)	38.6 (28.9, 48.3)	66.7 (57.7, 75.8)	28.1 (15.2, 41.0)	<0.0001
**At 10 Weeks**								
	**N=298**	**N=316**	**N=273**	**N=341**				
OPV 2	33 (10.9)	34 (8.5)	81 (29.9)	184 (53.5)	19.1 (10.1, 28.0)	45.0 (36.3, 53.7)	25.9 (13.8, 38.0)	0.0001
Penta 2	61 (20.2)	54 (14.6)	149 (54.8)	235 (68.4)	34.6 (26.2, 42.9)	53.7 (45.5, 61.9)	19.2 (7.8, 30.5)	0.0013
PCV 2	58 (18.8)	51 (13.7)	147 (54.1)	234 (68.2)	35.3 (27.7, 42.9)	54.5 (46.6, 62.4)	19.2 (8.5, 29.9)	0.0006
Rota Virus 2	57 (18.5)	51 (13.9)	148 (54.4)	234 (67.9)	35.9 (27.5, 44.3)	54.0 (45.8, 62.2)	18.1 (6.7, 29.5)	0.0023
**At 14 Weeks**								
	**N=288**	**N=303**	**N=266**	**N=330**				
OPV 3	24 (7.8)	29 (7.8)	68 (26.2)	153 (47.3)	18.5 (11.6, 25.4)	39.5 (29.7, 49.4)	21.1 (9.4, 32.7)	0.0006
Penta 3	24 (8.3)	29 (7.9)	53 (21.0)	125 (38.4)	12.7 (6.5, 18.9)	30.5 (21.6, 39.5)	17.8 (7.2, 28.4)	0.0013
PCV 3	24 (8.0)	29 (7.9)	53 (21.0)	124 (38.2)	12.9 (6.6, 19.3)	30.3 (21.4, 39.3)	17.4 (6.8, 28.0)	0.0018
IPV 1	61 (19.5)	59 (17.1)	148 (56.5)	228 (68.9)	37.1 (29.1, 45.1)	51.8 (41.8, 61.9)	14.7 (2.3, 27.2)	0.0212
**At 9 Months**								
	**N=249**	**N=256**	**N=237**	**N=275**				
Measles 1	56 (20.6)	56 (19.8)	154 (66.4)	228 (83.9)	45.8 (35.6, 56.0)	64.2 (53.9, 74.4)	18.4 (4.3, 32.5)	0.0111
**At 15 Months**								
	**N=186**	**N=191**	**N=181**	**N=189**				
Measles 2	24 (11.6)	28 (12.9)	62 (35.0)	87 (45.5)	23.4 (13.8, 33.0)	32.6 (18.1, 47.2)	9.2 (-7.8, 26.2)	0.2821

Table S3: Quetta - UC-Level Immunization Coverage at Baseline and Endline for Children Under-Three in Intervention and Control Areas

	Baseline		Endline		Control-% diff (95% CI)	Intervention-% diff (95% CI)	DID estimate-% diff (95% CI)	DID estimate-P value
	Control	Intervention	Control	Intervention				
**Immunization Status**								
	**N=468**	**N=465**	**N=304**	**N=337**				
Fully Immunized	52 (10.4)	122 (28.2)	10 (3.8)	160 (49.0)	-6.6 (-10.9, -2.3)	20.8 (10.9, 30.8)	27.4 (16.8, 38.0)	<0.0001
Partially Immunized	199 (42.0)	176 (37.8)	126 (42.4)	176 (50.8)	0.4 (-12.0, 12.9)	13.0 (2.7, 23.3)	12.6 (-3.3, 28.4)	0.1183
Non-recipients of Routine Immunization (NR-RI)	217 (47.6)	167 (34.1)	168 (53.8)	1 (0.2)	6.2 (-8.5, 20.9)	-33.8 (-41.8, -25.8)	-40.0 (-56.4, -23.6)	<0.0001
Zero Dose	212 (46.6)	165 (33.6)	15 (5.4)	1 (0.2)	-41.1 (-51.1, -31.2)	-33.4 (-41.4, -25.3)	7.8 (-4.8, 20.3)	0.2235
Ever had Vaccination Card	191 (39.5)	272 (60.7)	85 (29.0)	331 (98.3)	-10.5 (-23.0, 1.9)	37.6 (29.1, 46.0)	48.1 (33.3, 62.9)	<0.0001
**At Birth**								
	**N=468**	**N=465**	**N=304**	**N=337**				
BCG	252 (52.5)	300 (66.4)	133 (45.1)	309 (92.5)	-7.4 (-22.4, 7.6)	26.1 (17.7, 34.5)	33.5 (16.7, 50.4)	0.0002
OPV0	222 (46.6)	285 (63.0)	70 (24.2)	302 (90.5)	-22.4 (-34.9, -9.9)	27.5 (19.7, 35.3)	49.9 (35.5, 64.3)	<0.0001
**At 6 Weeks**								
	**N=457**	**N=459**	**N=304**	**N=330**				
OPV 1	80 (17.3)	193 (42.9)	66 (23.1)	249 (77.1)	5.8 (-5.9, 17.5)	34.2 (23.8, 44.6)	28.4 (13.0, 43.7)	0.0004
Penta 1	171 (37.5)	214 (48.5)	67 (23.4)	274 (84.4)	-14.1 (-26.0, -2.2)	35.9 (24.4, 47.4)	50.0 (33.8, 66.2)	<0.0001
PCV 1	160 (35.1)	208 (47.3)	67 (23.4)	273 (83.9)	-11.7 (-23.4, 0.0)	36.7 (25.1, 48.2)	48.3 (32.2, 64.5)	<0.0001
Rota Virus 1	153 (33.2)	205 (46.7)	67 (23.4)	259 (80.1)	-9.8 (-21.4, 1.7)	33.4 (21.4, 45.4)	43.2 (26.9, 59.6)	<0.0001
**At 10 Weeks**								
	**N=446**	**N=452**	**N=299**	**N=322**				
OPV 2	67 (14.5)	184 (42.2)	25 (9.4)	209 (66.3)	-5.2 (-12.0, 1.6)	24.1 (13.9, 34.3)	29.3 (17.2, 41.3)	<0.0001
Penta 2	68 (14.8)	179 (41.9)	61 (21.9)	221 (70.2)	7.1 (-4.4, 18.6)	28.3 (16.4, 40.2)	21.2 (5.0, 37.4)	0.0109
PCV 2	68 (14.8)	178 (41.7)	61 (21.9)	219 (69.3)	7.1 (-4.4, 18.6)	27.6 (15.8, 39.4)	20.6 (4.4, 36.7)	0.0133
Rota Virus 2	68 (14.8)	174 (40.8)	61 (21.9)	215 (68.0)	7.1 (-4.4, 18.6)	27.2 (15.4, 39.1)	20.1 (3.9, 36.4)	0.0155
**At 14 Weeks**								
	**N=435**	**N=440**	**N=293**	**N=307**				
OPV 3	60 (13.2)	145 (35.2)	12 (4.6)	175 (58.7)	-8.6 (-14.0, -3.2)	23.4 (12.7, 34.2)	32.0 (20.2, 43.8)	<0.0001
Penta 3	59 (13.0)	145 (35.2)	12 (4.6)	173 (57.7)	-8.4 (-13.8, -3.1)	22.5 (10.9, 34.1)	31.0 (18.5, 43.4)	<0.0001
PCV 3	59 (13.0)	137 (33.2)	12 (4.6)	172 (57.5)	-8.4 (-13.8, -3.1)	24.3 (13.2, 35.3)	32.7 (20.7, 44.7)	<0.0001
IPV 1	106 (23.5)	164 (39.5)	60 (21.5)	212 (70.8)	-2.0 (-13.0, 9.0)	31.3 (20.5, 42.1)	33.3 (18.2, 48.4)	<0.0001
**At 9 Months**								
	**N=356**	**N=363**	**N=267**	**N=259**				
Measles 1	76 (20.7)	150 (44.1)	61 (24.0)	180 (70.9)	3.3 (-7.6, 14.3)	26.8 (17.3, 36.3)	23.5 (9.3, 37.7)	0.0015
**At 15 Months**								
	**N=251**	**N=267**	**N=222**	**N=199**				
Measles 2	27 (10.4)	77 (30.5)	46 (21.6)	97 (48.9)	11.3 (-0.2, 22.8)	18.5 (6.8, 30.1)	7.2 (-8.9, 23.3)	0.3769

Table S4: Sociodemographic Factors Associated with Zero-Dose Children based o Logistic Regression for UCs in All Three Districts

	Zero Doser		Bivariate			Multivariate		
	No	Yes	OR	CI	P-value	OR	CI	P-value
	N=3314	N=929						
**Gender**								
Male	1697 (50.6%)	469 (49.3%)	Ref					
Female	1617 (49.4%)	460 (50.7%)	1.053	(0.892, 1.242)	0.542			
**Arm**								
Control	1589 (51.9%)	494 (66.7%)	1.853	(1.265, 2.714)	0.002	2.289	(1.625, 3.225)	<0.001
Intervention	1725 (48.1%)	435 (33.3%)	Ref					
**Maternal Education**								
No formal Schooling	2534 (74.7%)	848 (89.0%)	7.531	(4.562, 12.433)	<0.001	3.315	(1.959, 5.612)	<0.001
Primary	288 (11.3%)	56 (8.8%)	4.903	(2.372, 10.136)	<0.001	2.780	(1.407, 5.496)	0.003
Secondary or above	492 (14.0%)	25 (2.2%)	Ref					
**Socioeconomic status**								
Poorest	469 (8.1%)	305 (21.0%)	11.927	(6.200, 22.944)	<0.001	11.452	(5.802, 22.608)	<0.001
Poor	557 (15.1%)	282 (26.7%)	8.120	(4.272, 15.432)	<0.001	7.490	(3.911, 14.342)	<0.001
Middle	690 (27.2%)	210 (32.4%)	5.469	(2.818, 10.612)	<0.001	4.800	(2.486, 9.267)	<0.001
Rich	839 (28.9%)	99 (15.4%)	2.456	(1.292, 4.669)	0.006	2.339	(1.233, 4.438)	0.010
Richest	759 (20.7%)	33 (4.5%)	Ref					

Table S5: Sociodemographic Factors Associated with Non-Recipients of Routine Immunization (NR-RI) Children based on Logistic Regression for UCs in All Three Districts

	NR-RI		Bivariate			Multivariate		
	**No**	**Yes**	**OR**	**C.I**	**P-value**	**OR**	**C.I**	**P-value**
	N=3034	N=1209						
**Gender**								
Male	1556 (50.9%)	610 (48.9%)	Ref					
Female	1478 (49.1%)	599 (51.1%)	1.083	(0.944, 1.242)	0.255			
**Arm**								
Control	1348 (48.0%)	735 (72.6%)	2.881	(2.010, 4.131)	<0.001	3.880	(2.857, 5.269)	<0.001
Intervention	1686 (52.0%)	474 (27.4%)	Ref					
**Maternal Education**								
No formal Schooling	2268 (72.9%)	1114 (90.0%)	6.795	(4.205, 10.981)	<0.001	2.866	(1.794, 4.578)	<0.001
Primary	283 (12.1%)	61 (7.3%)	3.304	(1.660, 6.575)	0.001	1.777	(0.963, 3.279)	0.066
Secondary or above	483 (15.0%)	34 (2.7%)	Ref					
**Socioeconomic status**								
Poorest	397 (7.6%)	377 (19.0%)	11.348	(6.534, 19.709)	<0.001	13.657	(7.495, 24.886)	<0.001
Poor	494 (14.9%)	345 (24.5%)	7.486	(4.338, 12.917)	<0.001	8.296	(4.701, 14.643)	<0.001
Middle	579 (24.7%)	321 (37.2%)	6.825	(3.923, 11.874)	<0.001	6.991	(3.987, 12.260)	<0.001
Rich	817 (30.6%)	121 (14.4%)	2.136	(1.287, 3.547)	0.003	2.172	(1.289, 3.660)	0.004
Richest	747 (22.1%)	45 (4.9%)	Ref					

Table S6: Sociodemographic Factors Associated with Zero-Dose Children based on Logistic Regression for UCs in Districts Quetta and Lakki Marwat

	Zerodoser		Bivariate			Multivariate		
	No	Yes	OR	CI	P-value	OR	CI	P-value
	N=1988	N=908						
**Gender**								
Male	1042 (51.6%)	461 (49.6%)	Ref					
Female	946 (48.4%)	447 (50.4%)	1.08	(0.907,1.287)	0.384	-	-	-
**Arm**								
Control	906 (52.7%)	480 (66.6%)	1.79	(1.246,2.584)	0.002	1.79	(1.278,2.498)	0.001
Intervention	1082 (47.3%)	428 (33.4%)	Ref			Ref		
**Maternal Education**								
No formal Schooling	1669 (79.8%)	832 (89.4%)	3.04	(1.585,5.824)	0.001	2.04	(1.041,4.003)	0.038
Primary	243 (15.4%)	55 (8.8%)	1.55	(0.681,3.538)	0.293	1.64	(0.742,3.630)	0.219
Secondary or above	76 (4.8%)	21 (1.8%)	Ref			Ref		
**Socioeconomic status**								
Poorest	330 (8.9%)	206 (14.9%)	2.67	(1.648,4.314)	<0.001	2.85	(1.677,4.829)	<0.001
Poor	311 (8.7%)	218 (14.8%)	2.69	(1.706,4.238)	<0.001	2.66	(1.686,4.200)	<0.001
Middle	397 (19.8%)	191 (22.4%)	1.79	(1.178,2.714)	0.007	2.24	(1.436,3.502)	<0.001
Rich	458 (28.5%)	163 (26.4%)	1.46	(0.935,2.292)	0.096	1.58	(0.986,2.525)	0.057
Richest	492 (34.1%)	130 (21.5%)	Ref			Ref		

Table S7: Sociodemographic Factors Associated with Non-Recipients of Routine Immunization (NR-RI) Children based on Logistic Regression for UCs in Districts Quetta and Lakki Marwat

	NR-RI		Bivariate			Multivariate		
	No	Yes	OR	C.I	P-value	OR	C.I	P-value
	N=1707	N=1189						
**Gender**								
Male	899 (52.2%)	604 (49.2%)	Ref					
Female	808 (47.8%)	585 (50.8%)	1.13	(0.967,1.317)	0.123	-	-	-
**Arm**								
Control	663 (46.1%)	723 (73.0%)	3.16	(2.228,4.474)	<0.001	3.36	(2.485,4.554)	<0.001
Intervention	1044 (53.9%)	466 (27.0%)	Ref			Ref		
**Maternal Education**								
No formal Schooling	1404 (77.7%)	1097 (90.1%)	2.49	(1.334,4.639)	0.004	-	-	-
Primary	235 (17.4%)	63 (7.6%)	0.93	(0.432,2.009)	0.855	-	-	-
Secondary or above	68 (4.9%)	29 (2.3%)	Ref					
**Socioeconomic status**								
Poorest	280 (8.8%)	256 (13.5%)	2.43	(1.557,3.787)	<0.001	3.77	(2.305,6.151)	<0.001
Poor	259 (8.6%)	270 (13.4%)	2.47	(1.612,3.784)	<0.001	3.06	(2.006,4.658)	<0.001
Middle	360 (20.9%)	228 (20.1%)	1.53	(1.069,2.196)	0.02	2.43	(1.690,3.484)	<0.001
Rich	365 (26.0%)	256 (30.7%)	1.88	(1.290,2.732)	0.001	2.43	(1.684,3.507)	<0.001
Richest	443 (35.6%)	179 (22.4%)	Ref			Ref		
